# How Abstract (Non-embodied) Linguistic Representations Augment Cognitive Control

**DOI:** 10.3389/fpsyg.2020.01597

**Published:** 2020-07-15

**Authors:** Nikola A. Kompa, Jutta L. Mueller

**Affiliations:** ^1^Institute of Philosophy, University of Osnabrück, Osnabrück, Germany; ^2^Psycho/Neurolinguistics Group, Institute of Cognitive Science, University of Osnabrück, Osnabrück, Germany; ^3^Department of Linguistics, University of Vienna, Vienna, Austria

**Keywords:** embodiment, abstract representations, inner speech, cognitive control, labels

## Abstract

Recent scholarship emphasizes the scaffolding role of language for cognition. Language, it is claimed, is a cognition-enhancing niche (Clark, 2006), a programming tool for cognition (Lupyan and Bergen, 2016), even neuroenhancement (Dove, 2019) and augments cognitive functions such as memory, categorization, cognitive control, and meta-cognitive abilities (“thinking about thinking”). Yet, the notion that language enhances or augments cognition, and in particular, cognitive control does not easily fit in with embodied approaches to language processing, or so we will argue. Accounts aiming to explain how language enhances various cognitive functions often employ a notion of abstract representation. Yet, embodied approaches to language processing have it that language processing crucially, according to some accounts even exclusively, involves embodied, modality-specific, i.e., non-abstract representations. In coming to understand a particular phrase or sentence, a prior experience has to be simulated or reenacted. The representation thus activated is embodied (modality-specific) as sensorimotor regions of the brain are thereby recruited. In this paper, we will first discuss the notion of representation, clarify what it takes for a representation to be embodied or abstract, and distinguish between conceptual and (other) linguistic representations. We will then put forward a characterization of cognitive control and examine its representational infrastructure. The remainder of the paper will be devoted to arguing that language augments cognitive control. To that end, we will draw on two lines of research, which investigate how language augments cognitive control: (i) research on the availability of linguistic labels and (ii) research on the active usage of a linguistic code, specifically, in inner speech. Eventually, we will argue that the cognition-enhancing capacity of language can be explained once we assume that it provides us with (a) abstract, non-embodied representations and with (b) abstract, sparse linguistic representations that may serve as easy-to-manipulate placeholders for fully embodied or otherwise more detailed representations.

## Introduction

According to embodied approaches to language, comprehending a phrase or utterance requires that one activates embodied, modality-specific – as opposed to abstract – representations. At the same time, evidence is accumulating that language scaffolds impressive cognitive achievements. Language is said to enhance core cognitive functions such as memory, learning, or cognitive control. Yet, the notion that language augments cognition does not square well with embodied approaches to language processing as many explanations of how language enhances cognitive functions draw on the notion of an abstract representation^1^. Unfortunately, neither the notion of an embodied representation nor the notion of an abstract representation is particularly clear; an adequately thorough explication is missing. Moreover, the way in which language augments cognition is not very well understood either; what features of language prove beneficial?; and what are the underlying mechanisms? In what follows, we will first explicate the two notions of representation (abstract vs. embodied) and then examine how language might augment cognition. In order to move the problem onto more tractable ground, we will focus on the way in which the availability and use of a linguistic code enhances cognitive control; thus, all claims we make are confined to that domain. To that end, we will not only discuss embodied and abstract representations but also other types of “linguistic” representations and argue that the cognition-enhancing capacity of language is best explained on the assumption that it provides us with different types of abstract, sparse representations that can generate structure, reduce cognitive load, and increase computational power.

These considerations are primarily meant as a contribution to the debate on language and embodied cognition. But, although not directly addressing the controversial question of whether we think in natural language or whether natural language is constitutively involved in (some forms of) cognition (cf. e.g., Carruthers 2002, for review and discussion), they may nonetheless contribute to that debate as well, as the idea of linguistic representations underlying overt and inner speech may suggest a way in which to spell out the idea that cognition is – in some cases and to some extent at least – linguistic.

## Representations

### Embodied and Abstract Representations

The notion of representation or idea (as it was variously called) is notoriously hard to spell out. It has a long and distinguished history. While in antiquity (especially in Plato’s work), ideas were not meant to be subjective mental contents but rather immutable, ideal entities, in late antiquity, and the middle ages they began their career as key notions in semiotic and epistemological theorizing. When in modernity (Descartes is often said to be the founding father of modern representationalism), the notion of representation or idea became the heavy-duty notion that we are familiar with today (Perler and Haag, 2010), it was already afflicted with a few problems. Early on, the notion of idea was ambiguous, as ideas were commonly taken to be the vehicles as well as the contents of thought. Also, the notion of abstract idea favored by Locke and others did not fit in with a pictorial conception of idea. Yet, what could a plausible non-pictorial conception of idea (that nonetheless explains how ideas can be acquired in experience) look like? These (and many more) problems have been inherited by recent accounts.

Consequently, some contemporary authors claim that we can (and ought to) do without a notion of representation (understood as something that matches the content of conscious experience) altogether (cf. e.g., Noë and Thompson, 2004). Others still maintain the notion of abstract and thus amodal representation as a core ingredient of mental computations (Dove, 2009, 2011, 2016; Binder, 2016)^2^. This, in turn, is challenged by those who claim that abstract (amodal) representations are dispensable; all it takes are embodied (modality-specific or perceptual) representations (cf. e.g., Prinz 2002; for early critiques cf. Machery 2006, 2007; Mahon and Caramazza 2008). The latter debate is often framed in terms of how concepts or conceptual knowledge may be represented in the brain (Mahon and Hickok, 2016).

With this debate as a starting point, we will first explore what types of representation underlie language production and comprehension. Importantly, we will distinguish between *conceptual* (embodied and abstract) representations (encoding conceptual information) and *linguistic* representations that form the linguistic code itself at its various levels. In line with common usage (within philosophy and psychology), we take conceptual representations to encode (semantic) information about concepts (or categories) such as DOG or CHAIR. But then, some linguistic representations encode *conceptual-linguistic* information, i.e., information about linguistic concepts or categories (VERB, NOUN, and so on). Thus, strictly speaking, one ought to distinguish between what one might call “conceptual-semantic” and “conceptual-linguistic” representations. To keep things as simple as possible, we will nonetheless continue to speak of “conceptual representations” and “linguistic representations” (encoding linguistic information of various types, cf. section Linguistic Representations), unless more detail is required. The aim is to better understand the ways in which language and the various types of representations it affords us may prove cognitively beneficial and may be engaged during cognitive tasks. Eventually, we will argue that language, by providing us with various kinds of representations, enhances different cognitive functions (in particular cognitive control) in different ways. Therefore, in what follows, we will employ a rather thin and uncommitted notion of representation. A representation, as we will use the term, is a pattern of neural activity that fairly robustly encodes information and is thus sensitive to that type of information (which may range from concrete sensory input to generalizations over or abstractions from such input); furthermore, its cognitive role is (at least in part) grounded in the fact that it encodes this information.

It is a well-rehearsed point in the literature by now that sensorimotor areas are activated during language processing (cf. Meteyard et al., 2012; Mahon and Hickok 2016, for recent reviews). It has been observed, for example, that when people listen to sentences or phrases containing action verbs (such as “grasp” or “kick”), motor areas are activated (cf. e.g., Hauk et al., 2004; Pulvermuller, 2005; Aziz-Zadeh et al., 2006). More specifically, roughly the same region is activated when hearing the phrase “grasping the pen” and when seeing a video of a hand grasping a pen (Aziz-Zadeh et al., 2006). Barsalou speaks of “neural reuse” (Barsalou, 2016, p. 1129–1130) in these cases (cf. also Barsalou, 1999)^3^. It is claimed that in coming to understand a particular term or sentence, a prior experience has to be simulated or reenacted. The representation thus activated is embodied (modality-specific) insofar as specific sensorimotor regions of the brain are thereby activated (cf. e.g., Jirak et al., 2010, for review).

Yet, what exactly makes a representation embodied? That it is in a sensorimotor format, some say (Mahon, 2015; Mahon and Hickok, 2016). Yet, this notion raises further questions.

1. It raises the “important question of whether a simulation is sufficiently fine-grained to merit being called “embodied” rather than being some sort of an abstraction, even if that abstraction is originally grounded in a specific action or situation (Sanford, 2008, p. 189).” How much of an experience has to be embodied or simulated; how detailed does the simulation have to be? And, is not any sensorimotor representation an abstraction already (Mahon and Hickok, 2016)?
2. What exactly is the claim at issue? Is the claim that embodied representations are necessary (or even sufficient) for coming to understand a particular term (or grasping a particular concept)? Or is it rather the claim that embodied representations facilitate comprehension without being strictly necessary?^4^ Alternatively, some claim that they are simply epiphenomenal, mere by-products of linguistic or conceptual processing. Patient studies showing dissociation between concept possession (or linguistic comprehension) on the one hand and sensorimotor skills on the other speak against too tight a link between embodied representations (i.e., sensorimotor activation) and linguistic/conceptual understanding (cf. e.g., Mahon and Hickok 2016 for discussion). This suggests another, closely related question.
3. Are conceptual representations static and uniform or are they composed differently (or are different types of information drawn upon in a task-sensitive manner) across the variety of situations in which they are activated (Schyns et al., 1998; Vigliocco et al., 2004; Dove, 2016; Mahon and Hickok, 2016; Yee and Thompson-Schill, 2016)? An answer to this question depends on what we mean by “linguistic understanding.” The role of embodied representations in linguistic understanding can be adequately adjudicated only against the background of a theoretically sound model of what language understanding amounts to. An example may illustrate the point. Does a congenitally blind person grasp the meaning of the term “yellow” (or possess the concept *yellow*) in a similar manner as a normally sighted person? An answer to that question requires that we specify when understanding is achieved. If we agree that one understands a term if one is able to use it competently in different contexts, to draw valid inferences and to make correct judgments involving it, then we ought to answer the question in the positive (Saysani et al., 2018; Bedny et al., 2019). If, on the other hand, we require that a previous color experience is reenacted or simulated, then we ought to answer in the negative. But then, is not it the point of language that it allows speakers to acquire knowledge that goes beyond immediate sense experience, one might wonder (Dove, 2009, 2014; Binder, 2016)?

While the notion of embodied representation and its role in explaining language comprehension and production invites tricky questions, the notion of an abstract representation is no less problematic. For what is it for a representation to be abstract?

1. On a traditionally influential account, an idea becomes abstract by omission of distinguishing detail (Locke, 1979), thus by compressing information. For example, on seeing various persons, one abstracts from those aspects in which they differ and focuses only on what they have in common, thereby arriving at the (abstract) idea of a human being. As was already noted by contemporaries, this does not square well with a pictorial conception of ideas, as pictorial representations cannot omit detail *ad libitum*. The notion seems to fit better with a conception of ideas as lists of defining features. Yet, the presupposition that such a list is to be had for every idea seems problematic too. On a more recent account, abstraction is conceived of as transformational invariance, i.e., an increasing tolerance to slight transformations in the input (Buckner, 2018). This characterization is promising as it goes some way toward a functional characterization of what an abstract representation is. It tells us that the more abstract a representation is, the more it will tolerate somewhat transformed inputs.
2. But then, different types of abstract terms – and concepts or representations respectively – ought to be distinguished (Kompa, 2019). While every sort of classification requires abstraction, some terms (such as “red”) require that objects be classified according to sensory, *determinate* features. Others require that objects (or events) be classified according to *determinable* features; for example, the term “object” is applicable to entities that all have *a* shape, though not necessarily *the same* shape. Still others require that entities be sorted according to functional or defining features (“tool”), or according to evaluative features (“good”). And, still others require that entities be sorted according to structural or relational features (“being the same as”) or higher-order relational features, as when one judges that two pairs of objects have the same first-order relational property. While in all these cases, abstraction may be conceived of as increasing tolerance to transformations of the input in each there are certain features that can vary yet others that have to remain fixed. Most importantly, it is not just “simple,” determinate features (such as being a particular shade of red) that need to remain fixed but increasingly “complex” features manifesting a certain relational or evaluative structure. Unsurprisingly, then, the process of abstraction is often thought to result in hierarchies of increasingly abstract or complex representations. Also, integration and abstraction seem to go hand in hand. Mastery of evaluative terms, for example, consists in tolerating variation in the input while integrating information about a system of values and norms.

Early on, proponents of embodied accounts of concept representation discussed abstract concepts (Barsalou, 1999), carefully examined the content of abstract terms (Barsalou and Wiemer-Hastings, 2005), and increasingly stressed the diversity and heterogeneity of abstract terms (Borghi et al., 2018b, 2019), resulting in multiple representations views such as the words-as-tools (WAT) model (Borghi et al., 2019). Different types of abstract concepts (for mental, emotional, or metacognitive states, mathematical or physical entities, etc.) are distinguished and said to rely on different cognitive mechanisms (Borghi et al., 2018b; Desai et al., 2018). Barsalou and others also increasingly stress the need for different types of representations of conceptual information (Pulvermüller, 2013; Barsalou, 2016), including abstract or general representations. At the same time, more hierarchical models of abstract representations which are said to “arise from a process of hierarchical conjunctive coding” (Binder, 2016, p. 1098), i.e., exhibit sensitivity to particular combinations of inputs (ibid), are suggested. Furthermore, more and more authors emphasize the role of language in the processing and acquisition of abstract concepts (cf. e.g., Barsalou et al., 2012, who stress the role of linguistic forms; Borghi et al., 2018a, who briefly address the role of inner speech; or Lupyan and Winter, 2018, who discuss labels and the role of (a lack of) iconicity). For all that, a thorough and systematic account of different types of abstract concepts, the ways in which they are abstract as well as the cognitive infrastructure underlying their mastery is still pending.

1. Most importantly, representations (underlying language processing) can encode not only conceptual-semantic information (in a more embodied or more abstracted fashion) but also other types of linguistic information. They can, for example, encode – and be sensitive to – morpho-syntactic or phonetic information (cf. section Linguistic Representations). That may result in rather sparse, abstract, easy-to-compute representations which can act as placeholders or stand-ins for (maybe even as a sort of pointer to) more detailed, richer representations^5^. They could be thought of as a sort of interface that encodes only very little information itself but can activate associated (sensory-motor, evaluative, affective, etc.) information in a task-sensitive manner. Also, they may invite combination and can help generate structured representations.
2. Finally, one might distinguish abstract (amodal) from multimodal representations. While the former would be responsive to slightly transformed inputs, the latter would be responsive to inputs from various modalities (Fernandino et al., 2016). Thus, multimodal representations might share features with abstract representations, such as integration and tolerance to transformations in the input, and also share features with embodied representations by encoding highly modality-specific, concrete information.

In sum, abstract representations encode less detail than embodied, modality-specific representations. They may be sensitive to relational and otherwise more complex, abstract properties and tolerate various transformations of the input. And, they may be sensitive to different types of information. Being abstract and sparse, they ought to increase computational efficiency and come with low transfer costs (Machery, 2016) as well as help to avoid cognitive load problems (Dove, 2011). Most importantly, conceptual (semantic) representations (be they embodied or abstract) are not the only representations involved in language processing – a fact that is not sufficiently acknowledged in current debates on the topic, or so we will try to show. There are different types of linguistic representations, which encode – and are thus sensitive to – various types of information.

### Linguistic Representations

All models of language and language processing assume different types and levels of linguistic representation. Linguistic representations can encode sensory-motor as well as more abstract information. Many linguistic theories differentiate between two mental systems that are involved in language processing, i.e., the mental lexicon, as a storage system for words and the mental grammar, as the set of rules that specify how linguistic units are combined (Bloomfield, 1933; Chomsky, 1965; Garrett, 1976; Pinker, 1991). Others see in this dichotomy merely a descriptive tool and suggest dropping a strict two-system view when it comes to investigating the functional processes that form the basis of language (e.g., Jackendoff, 2007). As we are interested in the types of representations afforded to us by language, we will not take sides in this debate. Different theoretical accounts make different assumptions about the content and the functional and anatomical substrate of different representations and how different representational levels interact. Yet, there is a basic agreement that sound-level representations (phonology), representations of syntactic classes and operations (syntax), and representations of meaning or concepts (semantics) ought to be distinguished (Chomsky, 1957; Levelt, 1989; Jackendoff, 2007). Thus, it seems clear that language provides us with one or more levels of representation (in addition to conceptual-semantic representations) that could potentially feed into various cognitive processes.

First, as language is coded by sound (or script), there is a level representing the sensory content of the linguistic unit, i.e., phonetic or orthographic information. As speech sounds and their combinations are perceived in a language-specific manner (Miyawaki et al., 1975; Massaro and Cohen, 1983; Werker and Tees, 1984), we must have stored representations of phonemes and phonotactic regularities of our language(s). Further, it is assumed that there is *at least* one intermediate level of lexical representation between the representation of the single sounds and the conceptual level. Most models in fact assume two levels, the lemma level of representation, in which syntactic properties and meaning are specified (Levelt, 1989), and the lexeme level, in which the specific phonological form is laid down (Dell, 1986; Levelt, 1989; Caramazza and Miozzo, 1997). Whether there is an additional lemma level of representation assumed or not, it is uncontroversial that meaning-related, syntactic and phonological information about linguistic units can be accessed independently (Caramazza and Miozzo, 1997; Miozzo and Caramazza, 1997; Roelofs et al., 1998). Those models that do not assume a lemma level directly link semantic and syntactic information to the lexeme level (Caramazza and Miozzo, 1997; Miozzo and Caramazza, 1997). Despite these theoretical disagreements, it seems warranted to assume, following Jackendoff, that words are typically linked to phonological, syntactic, and conceptual-semantic levels of representation (Jackendoff, 2017, p. 193) as is illustrated for the word *cat* here:

- Phonology: /kæt/
- Syntax: +N
- Semantics: CAT

Note that those levels of representation might be very different from each other with respect to the richness, diversity, and structure of their content. Yet, for the current purpose, it is only important that they can be distinguished from each other and not so much how they are precisely characterized.

If we now adopt a view of representations as dynamic entities that are custom-built in a task dependent manner, it seems plausible to assume that language has the potential to provide its users with very different types of representations, depending on the task at hand. At times, this representational code may be sparse and stripped down to, e.g., morpho-syntactic information; at other times, it may be rich and include a whole wealth of conceptual-semantic (and maybe even pictorial or affective) information. More specifically, while lemma or morpho-syntactic representations are, presumably, rather on the abstract side (and while articulatory or motor representations are on the embodied side), phonological representations may be more or less abstract, depending on how much transformation in the input they tolerate. Also, this representational code may benefit from syntactic properties, which makes it easy to combine linguistic units into large and complex (relational) structures, supporting similar structures in other cognitive domains.

In the remainder of this paper, we would like to argue that these properties of representations afforded by language augment other domains of cognition, specifically cognitive control.

## Cognitive Control

Cognitive control (also termed executive functions) is an important set of processes in the service of optimizing behavior (Cohen, 2017, p. 16). It “is required for adaptive, goal-directed behaviors to solve novel problems, particularly those calling for the inhibition of automatic or established thoughts and responses” (Carlson and Beck, 2009, p. 163). At the very least, it comprises (cf. Cohen 2017 for an overview, and the contributions in Egner 2017 for some details):

- the ability to detect conflict and to resolve it through various gating mechanisms which result in the inhibition of prepotent, automatic responses.
- the ability to form, maintain, switch between and update internal goal representations in a task-sensitive manner.

While cognitive control is a well-established construct in psychology, its underlying mechanisms are still subject to debate. Neuropsychological, neurophysiological, and functional imaging research have associated cognitive control with the functions of the prefrontal cortex (Miller and Cohen, 2001). The type and interrelatedness of sub-functions, how exactly cognitive control is represented and computed in the brain, and the representational code of control signals are some of the questions still pending. Theories about cognitive control either focus on unifying, overarching principles, or on the distinctiveness of its sub-functions. The former assume domain-general, uniform principles explaining how various levels of cognitive control are supported by hierarchically organized operations of the prefrontal cortex (Christoff and Gabrieli, 2000; Miller and Cohen, 2001; Koechlin et al., 2003; Badre and D’Esposito, 2007). Yet, what these uniform principles might look like and how cognitive control may be supported by different sub-regions of prefrontal cortex along an anterior-to-posterior gradient is a topic of current debate. For example, it has been suggested that the temporal integration window of cognitive control (ranging from immediate stimulus processing to the integration of information about the past and the future; Koechlin et al., 2003) or the degree of abstraction in hierarchical action representations (Badre and D’Esposito, 2007) underlies the functional distinctions within the prefrontal cortex. Other theoretical approaches focus on the role and relation of the different distinguishable components of cognitive control. Miyake and colleagues (Miyake et al., 2000; Miyake and Friedman, 2012), for example, argue for the distinctiveness of three basic cognitive control operations, i.e., updating, flexibility, and inhibition. Barkley (2001), on the other hand, singles out non-verbal and verbal working memory, self-regulation of emotion, and reconstitution (i.e., flexibility) as core components of cognitive control. Arguably, both types of theories (those focusing on unifying principles and those focusing on sub-functions and domain-specific processes such as cognitive control in the language domain, cf. section The Availability of Labels as Facilitators of Cognitive Control) will help to improve our understanding of the neurocognitive organization of cognitive control (e.g., Jeon and Friederici, 2015; Badre and Nee, 2018).

The types of abstract representations that are accorded a role in hierarchical models of cognitive control are various and often hard to separate from each other by empirical means. Abstractness of representations with regard to prefrontal cortex function is said to result from: (i) domain generality (as opposed to domain specificity), (ii) relational complexity (indicating whether a response has to be sensitive to simple stimulus properties, to first-order, or higher-order relational properties), (iii) temporal abstraction (with response-selection being based on cues relating to different time scales), or (iv) generalization or governance (with abstract representations generalizing over or governing sets of more specific representations; Badre, 2008; Badre and Nee, 2018).

This leads us to questions about the representational infrastructure of cognitive control. The variety of domains that implement cognitive control and its efficiency with respect to novel tasks seems to demand a systematic, combinatorial code specifying the current control demands (Cohen, 2017). If such a code exists, it would be highly plausible that it shares some properties with language, specifically its capacity for abstraction and compositionality, as it needs to be able to work over arbitrary and novel content in similar ways. And even if such a general code supporting cognitive control does not exist, one has to consider that cognitive control processes have to deal with representations of various degrees of abstraction and complexity, ranging from motor sequences to the planning of future action goals. Thus, a cognitive control system must have at least the computational capacity to deal with a large degree of variability and abstraction. Biologically-based computational models have provided mechanisms that could, in principle, achieve symbolic-like computations in the regions involved in cognitive control (Rougier et al., 2005; Kriete et al., 2013). In the subsequent sections, we will explore whether and how language as an input code to this system could act as a booster. Some aspects of cognitive control may be uniquely human, as is the capacity for language. Potentially, this may be partly due to the way in which both systems are functionally integrated. In order to argue in favor of that point, we will bring together evidence suggesting that the availability of a linguistic code supports cognitive control and that active use of language, specifically inner speech, serves the same purpose. Crucially, we hold that it is not so much the embodied aspects of language but rather its abstract and combinatorial nature that is primarily responsible for the enhancement of cognitive control.

## The Availability of Labels as Facilitators of Cognitive Control

In various studies, it could be shown that different cognitive tasks and functions benefit from the availability of a linguistic code, especially from the availability of symbolic labels. Evidence stems from research on categorization, analogical reasoning, learning, memory, and cognitive control (Xu, 2002; Carlson et al., 2005; Lupyan, 2012; Althaus and Westermann, 2016; Doebel et al., 2018; Huang and Awh, 2018; LaTourrette and Waxman, 2019). For present purposes, we will zoom in on the role that symbolic labels may play for cognitive control. Labels can take on at least two roles here, depending on whether language (production) is the domain that has to be controlled or whether language is a means of controlling. In the former case, the cognitive domain that recruits control processes is linguistic. In the latter case, language (labels) represents operational aspects of the task, e.g., participants could use linguistic task cues such as “if red cube on right side of the screen press right button” to enhance performance. In this case, the task domain is visuo-spatial but the cognitive operation receives linguistic support.

We will treat the first role of labels only briefly, although cognitive control is involved in language processing at many levels ranging from language production to sentence comprehension and specific phenomena like code switching (Levelt, 1989; Hagoort, 2005; Bourguignon and Gracco, 2019; Sulpizio et al., 2020). It touches on the question of how closely linguistic and control systems are connected. We will focus here on whether cognitive control, when it is in the service of language-related tasks, is somehow different from cognitive control during non-linguistic tasks. Jeon and Friederici (2013, 2015) systematically investigated this question and compared linguistic and non-linguistic material with comparable affordances of hierarchical control. Participants were presented with hierarchically structured Korean symbols either with or without linguistic explanations. Both task conditions involved the anterior-to-posterior gradient of cognitive control in the prefrontal cortex (Jeon and Friederici, 2013). Yet, hierarchically structured sentences from the native language, i.e., highly familiar linguistic material, were processed by posterior prefrontal cortex (BA 44) only, even at a high level of hierarchical complexity. The authors argue that the high degree of automaticity that is typical of native language processing impacts on how the prefrontal cortex supports processes of hierarchical control (Jeon and Friederici, 2013). Thus, the idea is that the same type of formal (i.e., hierarchical) control demand engages different brain areas, depending on whether the task is novel or highly familiar (as is the case in natural language processing). This idea is in line with the view that brain areas supporting language processing are separable from those supporting domain general cognitive control (Fedorenko, 2014). Others argue for a more integrative view, in which language and domain general cognitive control are more intimately intertwined (Rouault and Koechlin, 2018; Bourguignon and Gracco, 2019). Differences between automatic and non-automatic language processing are here explained as differences along the temporal axis of cognitive control, whereby highly automatic language processing involves chunking processes within a single (not hierarchically structured) task-set while non-automatic linguistic processes are supposed to involve the generation of successive independent task-sets (Rouault and Koechlin, 2018). In a similar vein, an integrative view of language and cognitive control is supported by the observation that brain regions that are specialized in language processing and those that belong to domain-general control networks are closely linked during cognitive control in language production tasks (Bourguignon and Gracco, 2019)^6^

This brings us to the second role of labels and the question of how access to a linguistic code scaffolds cognitive control in non-linguistic tasks. Many studies using classical cognitive control tasks have revealed that performance is sensitive to the inclusion of symbolic representations in some aspects of the task. Several studies tested children’s performance in a reverse contingency task (in which participants have to point to the smaller of two rewards in order to receive the larger one) in the presence or absence of various types of symbolic labels substituting the real rewards. It was found that children performed better when labels were used (Carlson et al., 2005; Apperly and Carroll, 2009). Interestingly, the beneficial effect of labels does not entirely depend on the availability of a linguistic system, as similar effects were found in great apes (Boysen et al., 1996). Currently it is still unclear which property of symbolic labels causes this effect. It has been suggested that labels increase psychological distance in the face of an immediate reward (Carlson et al., 2005) or that they help to formulate alternative response strategies (Apperly and Carroll, 2009).

Another task that requires the inhibition of a prepotent response is the delay-of-gratification task. Participants have to reject an immediate reward in order to receive a larger reward later. This can be tested by either a choice task or a maintenance task. In the former, the delay cannot be influenced any more after the choice while the latter requires the suppression of the immediate reward for a longer period of time. It is long known that directing attention away from the arousing properties of the reward, e.g., by imagining the reward as a picture, makes it easier for young children to resist the immediate reward (cf. Mischel et al., 1989, for review). Some studies using delay-of-gratification choice tasks reported a reversed effect of symbolic labels, namely an increase of choices in favor of immediate rewards, observable in primates and human children (Addessi et al., 2014; Labuschagne et al., 2017). Yet, these results can also be explained as an effect of symbolic distancing: it is hypothesized that experiments with real food or food pictures may overestimate the abilities to tolerate delays in the participant as they might trigger impulsive choices due to the appetitive nature of the stimuli (Addessi et al., 2014). One of the most plausible explanations for the performance in these tasks seems to be that symbolic labels best sever the link between experience (stimulus) and response by activating abstract (“cool”) representations that are not too closely linked to the arousing (“hot”) aspects of the experience. Note that labels also impact cognitive control beyond delay-of-gratification or reverse-contingency tasks. It has been shown, for example, that 3-year olds benefitted from labeling in other cognitive control tasks, e.g., the dimensional card sorting task or complex visual search tasks (Kirkham et al., 2003; Miller and Marcovitch, 2011) although there are studies which could not replicate such effects (Müller et al., 2008). If labels were to only activate embodied, modality-specific representations, i.e., simulations of prior experiences, no psychological distance would come about. In line with this view, it has been argued that labels can, occasionally, “carry the burden of conceptual processing under a range of circumstances by effectively acting in place of deeper, more detailed representations of referent meaning” (Connell, 2019, p. 1308), especially in tasks that require only “shallow or superficial conceptual processing” (Connell, 2019, p. 1313). It therefore seems plausible that the representations engaged during those tasks are rather abstract. Kharitonova et al. (2009) and Kharitonova and Munakata (2011) provide direct evidence that participants who successfully perform in a switching task apply more abstract representations compared to less successful participants, as the former are also better in generalizing an acquired rule to novel items.

All these findings and considerations point toward a role for abstract linguistic representations as facilitators of cognitive control. If labels have such a role to play, one may expect that linguistic impairments may affect the performance in cognitive control tasks. This seems to be borne out by the available evidence. Aphasic patients and individuals with developmental language impairments have been shown to be somewhat impaired in cognitive flexibility and inhibition tasks (Baldo et al., 2005; Pauls and Archibald, 2016). Note, though, that there are also dissociations between linguistic and non-linguistic tasks in both aphasia and developmental language disorders, clearly supporting the view that language and complex cognition are not to be equated (Fedorenko and Varley 2016; Archibald 2017, for review). Furthermore, evidence from language impairments rather attests to the active (online) use of language in cognitive tasks (as compared to compensatory strategies) than to the internal (offline) availability of a linguistic code, which is difficult to assess in those cases. These strategic uses of language for the purpose of formulation of cognitive control task affordances will be treated in more detail in the following section.

## The Use of Inner Speech in Support of Cognitive Control

At the beginning of the 20th century, Vygotski (1986) propagated the notion that inner speech is internalized public speech and retains some features of the latter (e.g., a social aspect), while losing others (e.g., by being compressed). He claimed that when acquiring a language, the child first talks out loud in what he called, following Piaget, “egocentric” speech, and what is today called “private speech.” In the course of development, speech is more and more directed at the child themself, and private speech slowly transforms into inner speech. Inner speech is inaudible to others (Alderson-Day and Fernyhough, 2015, p. 931). The speaker “apprehends him or herself to be speaking meaningfully without producing any accompanying sound or appreciable bodily […] movement (Hurlburt et al., 2013, p. 1482)^7^.”

Different things go by the name “inner speech” though. Inner speech ought to be distinguished from the auditory imagery of speech (Machery, 2005; Hurlburt et al., 2013; Gauker, 2018) or inner hearing (Fernyhough, 2016), although in conscious inner speaking, one seems to always accompany the other. It ought to also be distinguished from “unsymbolized” thinking (Hurlburt and Akhter, 2008), although there may be a gradient from fully explicit, articulate (if unarticulated) inner speech to compressed, truncated (still language-based) thinking (that is no longer experienced as “speech,” lacking a recognizable phonetic profile). One might hypothesize that the latter still activates (something like) lemma representations but no longer activates phonological or articulatory representations.

Moreover, inner speech is put to different uses and serves different ends. It may occur while one is engaged in a cognitively demanding task, as when one is reflecting on a problem, planning an action, or deliberating more generally; it may take the form of an inner monologue or dialogue (Fernyhough, 2009). It may take the form of self-regulatory and also motivational self-talk, as when one preps oneself for a sporting performance (in which one often addresses oneself as “you”; cf. Fernyhough 2016). One engages in inner speech while silently rehearsing something and also when one is daydreaming, letting one’s mind wander (Wiley, 2016). It allows us to think about thoughts, being, arguably, “the single most important tool for intentional ascent” (Bermudez, 2018, p. 204); and so on and so forth.

Most importantly for our purposes, there is an ever-growing body of evidence supporting the notion that private or inner speech enhances children’s (and to a lesser extent adults’) performance in different memory, planning, and problem-solving tasks (Diaz and Berg, 1992; Winsler et al., 2009). Evidence is accumulating that inner speech enhances cognitive flexibility by aiding retrieval and activation of task goals (Miyake et al., 2004).

It has been shown that verbal self-instructions improves performance in switching tasks, especially in children and the elderly (Kray et al., 2008). One of the most famous examples of a switching task is the Wisconsin Card Sorting Paradigm, in which children are asked to sort bivalent cards (e.g., green boats, red boats, green cows, and red cows) first according to one dimension (e.g., shape) and are then asked to sort along the other dimension (color). In a similar vein, switching costs have shown to increase in adults when inner speech is disrupted, e.g., *via* articulatory suppression (Emerson and Miyake, 2003; Miyake et al., 2004). Recently, a role for inner speech in task switching has also been shown in an interference-free setting, *via* electromyographic recordings from the tongue (Laurent et al., 2016). Yet, it is not only flexibility that is affected by overt or coverts verbalization but also other aspects of control tasks such as inhibition (Kray et al., 2009), task maintenance (Saeki et al., 2013), and control focus (proactive vs. reactive control; Kray et al., 2015) have been shown to be modified by task-related verbalizations. While these examples highlight the function of inner speech as an additional tool for coding task-related representations that are used during task processing, there is further evidence that even evaluative and motivational inner speech that does not directly represent the task can enhance performance in classic cognitive control tasks. Gade and Paelecke (2019) found that participants who reported the habitual use of motivational and evaluative inner speech showed less conflict in two classic cognitive control tasks (the Simon and Flanker tasks). Consistent with these findings, recent reviews conclude that inner speech, while maybe not strictly necessary, nonetheless augments different aspects of cognitive control (Cragg and Nation, 2010; Kray and Ferdinand, 2013).

Especially in cognitively demanding tasks requiring high levels of control, inner speech may help to represent task-related information and to retrieve, maintain, update, and manipulate task representations. The linguistic representations provided by language may serve as a good proxy in order to quickly build or modify abstract control representations. If language was such a support system (instead of an integral component of the control system), one should expect a positive impact of language specifically for unpracticed, novel tasks. Language could serve as an important function in formulating task representations but become superfluous once those representations were installed. A recent study by van’t Wout and Jarrold (2020) confirms this intuition by reporting articulatory suppression effects during the initial phase of novel task learning and not in a later phase. Support may also come from studies on rapid instructed task learning (RITL, for short), i.e., “the ability to learn task procedures from instruction” (Cole et al., 2013, p. 1), an “especially important form of cognitive flexibility” (Cole et al., 2013, p. 1) and something humans – as opposed to other animals – excel at. And, while language does not seem to be strictly necessary, and although limited RITL-abilities have also been found in monkeys and non-human primates, RITL that employs linguistic means seems to be “the most powerful form” (Cole et al., 2013, p. 3). This may be due to the fact that it increases high-fidelity transmission of task-relevant information. But, again, one might also hypothesize that language not only helps to formulate task instructions in overt speech but also to come up with and maintain (increasingly abstract and less context-bound) task rules in inner speech. Also, integrative models of RITL highlight the combinatorial properties of the representations underlying task learning and the resulting cognitive flexibility (cf. Cole et al., 2013, for discussion), something linguistic (e.g., lemma) representations could deliver.

All in all, it seems that inner speech influences performance in cognitive control tasks through several mechanisms. At times, it may be useful for the representations of task-related aspects. The abstract and sparse linguistic code may aid memory retrieval, maintenance, and manipulation of task representations. Such computational benefits are easier to explain when taking into account the combinatorial properties of (abstract) linguistic representations (as opposed to embodied ones). At other times, when inner speech improves performance as motivational self-talk, the psychological distancing function of language may come to the fore with inner speech also helping to monitor one’s performance and to ensure that one stays on task. Thus, there is probably no unitary function of inner speech that improves cognitive control but rather several aspects of it that, nonetheless, all serve to enhance the uniquely human power of cognitive control.

## Discussion and Open Questions

The following picture emerges. Once one asks what types of representations are activated during linguistic processing, it becomes clear that one ought to distinguish (at least) between articulatory/motor, phonological, morpho-syntactic/lemma, and conceptual representations. The question of what conceptual representations are and how concepts are represented in the brain has garnered a lot of attention within philosophy and cognitive science and fuels the controversy between those who claim that conceptual representations are necessarily embodied and those who deny it. The cognitive potency and function of these other linguistic representations are less discussed in the literature.

Language unquestionably affords us cognitive benefits. Some of these benefits, we argue, are best explained on the assumption that language provides us with abstract and sparse representation. As outlined above, the availability and active usage of a linguistic code have been shown to enhance cognitive control. Plausible mechanisms of how language in general, and labels in particular, aid cognitive control are the increase of psychological distance by, arguably, activating abstract representations not immediately bound to action or perception. Those representations could encompass linguistic representations beyond conceptual ones, as, for example, abstract lexical (lemma) or phonological representations. Furthermore, the linguistic code is sparse and thus computationally cheap, yet powerful, as it exhibits combinatorial structure. Due to these properties, it may help to formulate, maintain, retrieve, and switch between task rules (“If stimulus X appears, then act in manner Y”). We conjecture that this is the basis of the cognitive functions of inner speech: based on the computational advantage of linguistic representations, inner speech enhances performance in problem-solving and other cognitively demanding tasks and augments cognitive control more generally.

The representational infrastructure of language, in overt or covert (inner) speech, consists of phonological, abstract-lexical, and syntactic representations, which may or may not be accompanied by embodied representations. The representations supporting cognitive control functions also seem to involve various kinds of abstraction. Assuming that representations with similar informational content are easier to map onto each other than to representations including different degrees of detail, it seems plausible that especially the more abstract properties of the linguistic code feed into the system guiding cognitive control.

All this is not to deny that detailed, embodied, sensory-motor representations may be of use, too. They may, occasionally, lead to deeper memory encoding, better retrieval, better multimodal processing, etc. Social cognition may also benefit from embodied linguistic representations as they may allow speakers to mentally align more easily by simulating similar experiences. They may also ease language acquisition and in many cases, language comprehension. Interpreting a novel metaphor or a poem, for example, may require that very rich, detailed, sensory-motor or affective representations are activated in order to understand the particular aspects of meaning that are targeted. For all that, the cognitive benefits of less embodied, abstract, and sparse representations are not to be denied either (Kompa, 2019). In the end, a more balanced and nuanced view that acknowledges that (i) multiple (types of) representations may be activated and drawn upon in a task-sensitive manner in linguistic processing and that (ii) there may be a gradient ranging from more embodied to more abstract (and maybe to different types of abstract) representations which all play (different) cognitive roles, may be the most promising route.

Also, note that we are not inferring the linguistic character of (some forms of) cognition from the experience of inner speech. It has been argued (Machery, 2005) that the phenomenology of inner speech does not provide evidence for the claim that cognition is linguistic, as the latter claim concerns the vehicles of thought (or the types of representations grounding conscious experience), which are not consciously accessibly. All we are claiming is that the findings of studies examining the cognitive benefits of inner speech seem to be best explained – at least as far as its effect on cognitive control is concerned – on the assumption that inner speech activates abstract linguistic representations of sorts. It is not an inference from phenomenology to neural implementation (that would indeed be invalid) but an inference to the best explanation of some of our cognitive accomplishments. Still, one might wonder whether those linguistic representations (allegedly) activated during control-demanding tasks (or in inner speech, for that matter) are consciously accessible. Now, while lemma or morpho-syntactic representations do not seem to reach the level of consciousness, phonological representations may (but need not) do so. But then, there might be ways in which lemma representations (or some such thing) can be experienced, as suggested by studies on tip-of-the-tongue phenomena (cf. e.g., Vigliocco et al., 1997). This is slightly at odds with Carruther’s claim that conscious access “always depends on attention directed at sensory representations of some sort” (Carruthers, 2018, p. 39). He argues that “most inner speech results from the mental rehearsal of speech actions” (Carruthers, 2018, p. 33), thus also involving the speech production system. In inner speech episodes, we activate but do not execute speech actions, and “[t]hese motor schemata are used to create a representation of what it would sound like if they were carried through to completion” (Carruthers, 2018, p. 33). On our view, executing may be stopped much earlier, maybe even before phonological representations become activated (thus at the level of lemma representations). Moreover, Carruthers has it that inner speech, being but a “copy of motor instructions” (Carruthers, 2018, p. 43), has no semantic content and needs to be interpreted by the speech comprehension system. This strikes us as a problematic idea, for why would one want to activate a speech action in inner speech that completely lacks content? What would be the point of that?

Finally, while the general conclusion that language aids cognitive control seems warranted, we also acknowledge that there are many open research questions with regard to how this comes about. For example, it is still not clear which properties of linguistic labels are responsible for their cognitive potency: is it their familiarity, their referential function, their phonological profile, their non-iconicity (i.e., the fact that they do not resemble what they denote), or something else still? Also, how do inner speech and outer speech relate to one another; what form can inner speech take and which purposes (over and above those indicated here) does it fulfill? How exactly are syntactic properties and combinatorial abilities implemented so as to mirror complex task structures? How exactly do lemma, conceptual, phonological, and other linguistic representations relate to one another with regard to their function for cognitive control? How do other cognitive domains, like memory, interact with language in the service of cognitive control? Does language play similar roles across different cognitive domains, e.g., cognitive control, memory, and learning? Future research will have to tackle these questions and will, hopefully, lead toward more detailed, explanatory models of how language and cognition interact.

## Author Contributions

The authors developed the ideas presented in the paper in close collaboration and as a result of intensive discussions on the topic. All authors contributed to the article and approved the submitted version.

### Conflict of Interest

The authors declare that the research was conducted in the absence of any commercial or financial relationships that could be construed as a potential conflict of interest.

## Footnotes

^1^The position defended here is incompatible with embodied accounts that claim that linguistic processing necessarily and predominantly recruits sensory-modal areas in the brain. More moderate, hybrid accounts that acknowledge a role for abstract representations and allow for more flexible activation of different types of representations in linguistic processing are compatible with our view (see section Discussion and Open Questions).

^2^Dove, in fact, defends a hybrid view, arguing that we need embodied as well as dis-embodied representations (Dove, 2011). He has it that language gives us “access to a new type of representational format” (Dove, 2014, p. 373). It is “an external symbol system – one that has the computational features associated with amodal symbol systems – that we learn to manipulate in an embodied and grounded way” (Dove, 2018, p. 1).

^3^The idea of neural reuse is developed in detail by Anderson, who suggests that “the brain achieves its variety of function by using the same regions in a variety of circumstances, putting them together in different patterns of functional cooperation” (Anderson, 2014, p. 5; cf. also Anderson, 2010).

^4^These two options do not exhaust the space of possibilities. Embodied representations could be causally relevant without being causally necessary, as something else might play the causal role too. Moreover, embodied representations could be constitutive of comprehension (in that they would have to figure in a mechanistic explanation) as opposed to being causally necessary (comprehension might be counterfactually dependent on embodied representations).

^5^Of course, we do not originate this idea (cf. e.g., Barsalou 2016, p. 1134 for a brief review); yet often, existing accounts do not bother to spell out in detail what they mean by “linguistic representation.”

^6^Also, whether cognitive control processes during language processing are language-specific or not, linguistically coded semantic knowledge may provide an additional control system that can be exploited by non-linguistic domains of cognition, termed “semantic control” (cf. Lambon Ralph et al., 2017; Bourguignon and Gracco 2019, for review).

^7^Empirically investigating inner speech raises tricky methodological questions and seems to call for a methodologically pluralist approach. Unsurprisingly, then, there are neuroimaging and neuropsychological studies examining the neural correlates of inner speech; others devise questionnaires or engage in descriptive experience sampling (cf. Alderson-Day and Fernyhough 2015, for review).
